# Utility of PPO-FEV1%pred in predicting postoperative pulmonary complications after secondary surgery in patients with multiple primary lung cancers

**DOI:** 10.3389/fonc.2026.1747652

**Published:** 2026-02-04

**Authors:** Yifan Wang, Shihao Shao, Yun Sha, Zhenchao Lv, Qingna Liang, Jing Peng, Yong Fei, Zhonghui Wang, Jinyuan Yang

**Affiliations:** 1 Department of Anesthesiology, Yunnan Cancer Hospital, The Third Affiliated Hospital of Kunming Medical University, Peking University Cancer Hospital Yunnan, Kunming, China; 2 Department of Radiology, Yunnan Cancer Hospital, the Third Affiliated Hospital of Kunming Medical University, Peking University Cancer Hospital Yunnan, Kunming, China

**Keywords:** postoperative pulmonary complications (PPCs), predict model, pulmonary function test (PFT), quantitative computed tomography (QCT), secondary pulmonary resection

## Abstract

**Objective:**

This study aimed to determine the predictors of postoperative pulmonary complications (PPCs) in patients undergoing secondary pulmonary resection for multiple primary lung cancers (MPLC), thus facilitating targeted clinical management strategies.

**Methods:**

Clinical and computed tomography (CT) imaging data from MPLC patients treated at the Third Affiliated Hospital of Kunming Medical University between January 2022 and June 2023 were retrospectively analyzed. Participants were categorized into PPC and non-PPC cohorts. Initially, univariate analyses were conducted to assess clinical characteristics and CT parameters that significantly differed between groups. Subsequently, Independent predictors were identified via multivariate logistic regression. Receiver operating characteristic (ROC) curve analysis was performed to evaluate diagnostic performance of the identified variables. Internal validation was performed using bootstrap resampling (1,000 resamples), and calibration was assessed using a calibration plot with goodness-of-fit testing. In addition, two prediction models were developed, including a pre-/intraoperative model (Model 1) and an early postoperative augmented model (Model 2); sensitivity analyses were conducted by excluding minor PPC events.

**Results:**

A total of 170 patients were included, with postoperative pulmonary complications (PPCs) occurring in 52 cases. Univariate analysis revealed no significant differences between the PPC and non-PPC groups in terms of gender, smoking index, preoperative PaO_2_, operative duration, single-lung ventilation time, moderate postoperative pain at 48 hours, closed thoracic drainage duration, FEV1%pred, low attenuation volume percentage (LAV%), resected functional lung volume (RFLV), PPO-FVC, PPO-FEV1, PPO-MVV, PPO-FEV1%pred, PPO-MVV%pred, and PPO-DLCO%pred (P<0.05). Multivariate logistic regression analysis indicated that FEV1%pred (OR = 0.86, 95% CI: 0.789–0.938), unilateral ventilation duration (OR = 1.009, 95% CI: 1.000–1.019), LAV% (OR = 1.057, 95% CI: 1.011–1.106), moderate pain at 48 hours postoperatively (OR = 12.32, 95% CI: 3.903–38.898), and PPO-FEV1%pred (OR = 0.86, 95% CI: 0.789–0.938) were independent predictors of PPCs. In single-factor ROC analysis, PPO-FEV1%pred demonstrated optimal discriminatory ability (AUC = 0.801, cutoff value 79.45%). Regarding model construction: - The preoperative/intraoperative model (Model 1) demonstrated an AUC of 0.86 (95% CI: 0.80–0.92) after bootstrap internal validation (1,000 iterations), with a calibration slope of 0.93 and a Hosmer–Lemeshow test P = 0.466; The early postoperative enhancement model (Model 2) yielded an AUC of 0.91 (95% CI: 0.87–0.96), calibrated slope of 0.90, and Hosmer–Lemeshow test P = 0.203. Sensitivity analysis (excluding minor events) demonstrated that PPO-FEV1%pred retained the strongest discriminatory capacity (AUC = 0.780, 95% CI: 0.696–0.863; cutoff value 78.94%; sensitivity 80.95%; specificity 67.86%), indicating robustness of the primary outcome.

**Conclusions:**

This study identified FEV1%pred, one-lung ventilation time, LAV%, PPO-FEV1%pred, and moderate pain at 48 h postoperatively as independent predictors for PPCs. PPO-FEV1%pred demonstrated the highest diagnostic accuracy in predicting PPCs after secondary pulmonary resection, facilitating personalized clinical decision-making and patient management. Findings remained robust in sensitivity analyses.

## Introduction

Based on the data reported in 2022, lung cancer continues to represent the most common cause of cancer-related morbidity and mortality within China (1). With the increasing application of low-dose spiral CT, the detection rate of multiple, small pulmonary nodules has significantly risen (2). Accumulating evidence indicates that the presence of these multiple nodules frequently corresponds to MPLC (3). Surgical resection remains the primary treatment for multiple primary lung adenocarcinomas (4). Due to poor patient tolerance for synchronous surgery, secondary surgery is currently preferred by most surgeons (5). However, previous studies indicate that secondary surgery significantly increases the risk of PPCs and patient mortality (6). Therefore, assessment to predict PPCs is crucial. Nevertheless, no previous studies have addressed this issue, making it critically important to develop a simple and accurate evaluation method.

Perioperative pulmonary function testing is crucial for identifying pulmonary complications (7–9). Kocher et al. reported that dynamic lung function assessment effectively predicts postoperative pulmonary complications (7). Li et al. also showed that perioperative respiratory monitoring significantly correlates with PPC (8). Brunelli et al. demonstrated that pulmonary function parameters are essential for PPC risk stratification (9). Some scholars have pointed out that the estimation and analysis of postoperative predicted forced expiratory volume in the first second (PPO-FEV1) and postoperative predicted diffusing capacity for carbon monoxide (PPO-DLCO) are significantly correlated with the occurrence of PPCs (10). However, the segment-based formulas for calculating PPO-FEV1 and PPO-DLCO assume uniform functional distribution across all lung segments. Due to compensatory changes in the remaining lung tissue after initial surgery, uniform functional distribution among segments is difficult to achieve in patients undergoing secondary surgery. Thus, new methods are required to evaluate preoperative pulmonary function in patients undergoing multiple surgeries. Previous studies suggested that computed tomography could assess functional lung units, enabling more accurate prediction of PPO-FEV1 and PPO-DLCO (11, 12).

Quantitative computed tomography (QCT) employs specialized quantitative CT software to precisely measure and analyze conventional CT scan data. Through standardized acquisition of thoracic CT images and automated segmentation techniques, QCT enables quantitative assessment of parameters such as lung volume, parenchymal density, airway and pulmonary vessel diameters, and wall thickness (13). Dedicated QCT pulmonary function analysis software facilitates quantitative evaluation of lung function either for the entire lung or specific lobes.

However, the predictive efficacy of QCT for PPCs following secondary thoracic surgery remains uncertain. Moreover, the potential correlation between patients’ clinical characteristics and the incidence of complications is unclear. Given these knowledge gaps, this study aims to identify reliable predictive indicators for PPCs after secondary surgery, providing evidence-based guidance for clinical decision-making and patient management.

## Materials and methods

### Patients

This retrospective study involved clinical records from lung cancer patients admitted to the Third Affiliated Hospital of Kunming Medical University(Yunnan Cancer Hospital) between January 2022 and May 2023, who were candidates for secondary thoracic surgery. The inclusion criteria were: (1) patients diagnosed with synchronous MPLCs scheduled for secondary thoracic surgery; (2) patients with metachronous MPLCs identified during post-resection surveillance and planned for secondary surgery; and (3) patients who underwent complete preoperative PFTs and CT examinations. The exclusion criteria were: (1) patients with preoperative pulmonary infection; (2) patients with significant preoperative pleural effusion; (3) individuals with severe thoracic deformities; (4) patients complicated by severe cardiac, hepatic, or renal dysfunction; and (5) cases with incomplete clinical records. Ethical approval for this study was granted by the Medical Ethics Committee of the Third Affiliated Hospital of Kunming Medical University(Yunnan Cancer Hospital) (Approval No. KYLX2023012). All consecutive patients undergoing secondary pulmonary resection during the study period were screened. Patients were excluded only according to the predefined exclusion criteria, without additional exclusions.

### PFTs

PFTs were performed using the MasterScreen lung function tester (CareFusion, Germany). Patients were seated upright with the nose clamped and breathed calmly or rapidly through the mouth according to instructions. Collected PFT parameters comprised forced expiratory volume in the first second (FEV1), FVC, FEV1/FVC ratio, maximal voluntary ventilation (MVV), and diffusing capacity for carbon monoxide (DLCO). The interval between CT imaging and PFT assessments did not exceed one week for all included patients.

### CT examinations

Chest CT imaging was performed using a Siemens Definition AS + 128-slice spiral CT scanner. Patients underwent scans in a resting state; detailed scanning procedures were explained in advance, including breath-holding exercises and deep breathing training. During the scanning process, patients were placed supine, arms elevated with hands positioned above the head, and instructed to maintain breath-holding following deep inspiration. Scanning extended continuously from the lung apices to the bases. Scanning parameters included automatic control of tube voltage and current, a reference current of 100 mAs, collimation width of 0.6 mm, rotation time of 0.5 s, and a pitch factor of 1.2. Lung window images were reconstructed using the SAFIRE iterative algorithm, employing a reconstruction thickness of 1 mm, a spacing of 0.75 mm, and a matrix dimension of 512 × 512. Contrast agents were not utilized during scanning.

### CT image analyses

CT images were imported into the Siemens syngo.via post-processing workstation in DICOM format and analyzed using CTpulmo 3D software. In this study, functional lung tissue was defined as tissue with attenuation values ranging from -910 HU to -600 HU. The software automatically segmented lung tissue within this interval to calculate total lung volume and density. The software also automatically identified and excluded large bronchovascular bundles and mass lesions from the analysis. Lung tissue intended for surgical resection was manually outlined using the software to evaluate lung density and volume. TFIV, emphysema index (LAV%), and mean lung density (MLD) were then calculated.

### Outlining the extent of lung tissue to be resected

#### Total lung resection (one side)

The affected side of the lung was automatically outlined using the Pulmo software. Bronchial and vascular bundles above the level of lung segments unrecognizable by the software were manually outlined and excluded from the assessment.

#### Lung lobectomy and segmental resection

For lobectomy, the software automatically outlined the interlobar fissure and arterial and venous routes of the lung segments. Segmental resections were manually outlined based on these criteria.

#### Lung wedge resection

Lung wedge resection typically involves removing lung tissue approximately 3–5 cm above and below the lesion; clinically, a resection range of about 3 cm is generally adopted. Thus, an area within 3 cm above and below the lesion was outlined, and the volume of the lobe containing the lesion within this area was calculated.

### Prediction of postoperative lung function

Predicted postoperative pulmonary function (PPO) was computed according to the formula: preoperative pulmonary function parameter × [1 − (RFLV/TFLV)]. Preoperative pulmonary function measurements and quantitative CT (QCT)-derived functional lung volumes (FLV) were input into this calculation. Resultant computed parameters included predicted postoperative maximal voluntary ventilation (PPO-MVV), predicted postoperative forced expiratory volume in the first second (PPO-FEV1), and predicted postoperative diffusing capacity for carbon monoxide (PPO-DLCO).

### Postoperative complications

PPCs were defined as thoracic or lung-related complications occurring within 30 days after surgery. The diagnostic criteria are presented in **Table 1** (14, 15). PPCs were analyzed as a composite endpoint (yes/no) to reflect overall clinically relevant pulmonary morbidity after secondary pulmonary resection. Postoperative pain was assessed at 48 hours post-surgery using the VAS, with moderate pain defined as VAS scores ≥4. Prolonged air leak (PAL) was defined as an air leak persisting for more than 7 postoperative days, consistent with prior thoracic surgery literature; we selected this stricter threshold to capture clinically meaningful air leaks that typically require prolonged pleural drainage and extend hospitalization (16, 17).

**Table 1:** Diagnostic criteria for PPCs.

PPCs	Diagnostic criteria
Pulmonary infection	(1) Fever >38 °C occurring 72 h after surgery; (2) Elevated white blood cells (>12×10^9^/L) or an increase >10×10^9^/L after returning to normal; (3) Chest imaging showing progressive increase in plate-like hyperdense shadows or solid lung lesions; (4) Purulent sputum or positive sputum culture. ≥3 criteria required; if (4) is positive, only one additional criterion needed
Pulmonary atelectasis	Partial or total lung atelectasis confirmed by imaging, typically diagnosed during fiberoptic bronchoscopic aspiration therapy
Prolonged air leak	Persistent air leak from the pleural drainage system persisting for >7 postoperative days, requiring ongoing pleural drainage and prolonged hospitalization.
Pulmonary embolism	Clinically symptomatic embolism diagnosed via CT and/or CT angiography (CTA)
Pneumothorax	Pneumothorax confirmed by imaging; ineffective simple thoracentesis requiring repeated closed chest drainage with a chest tube
Bronchospasms	Symptoms such as shortness of breath, bilateral lung rales, prolonged expiratory phase, or increased airway pressure during mechanical ventilation, relieved only by suction, expectoration, or bronchodilators
Pleural effusion	Clinical symptoms necessitating multiple thoracenteses or repeated closed chest drainage
Thoracic hemorrhage	Bleeding requiring surgical re-exploration or transfusion of ≥4U concentrated red blood cell suspension

### Statistical analyses

Continuous variables that conformed to normal distributions were analyzed by independent-sample t-tests, while variables not meeting normality assumptions underwent Mann–Whitney U testing. Categorical data were compared using Pearson’s chi-square test. Subsequently, backward elimination logistic regression analysis was employed to develop a predictive model. Variables were initially screened via univariate analysis. Clinically relevant variables and those showing statistical significance in univariate comparisons were entered into a multivariable logistic regression model. Backward elimination was used to construct the final parsimonious model. Statistical evaluations were conducted using SPSS (version 27.0). Optimal cutoff thresholds were established according to the highest Youden’s index. Receiver operating characteristic (ROC) curves were constructed with MedCalc software (version 20.217). The diagnostic efficiency of each candidate predictor was quantified by the area under the ROC curve (AUC). Statistical significance was defined as a P-value less than 0.05. Model performance was evaluated in terms of discrimination and calibration. Internal validation was performed using bootstrap resampling (1,000 resamples) to obtain an optimism-corrected AUC and an optimism-corrected calibration slope. Calibration was assessed using a calibration plot comparing predicted and observed probabilities, and model goodness-of-fit was additionally evaluated using the Hosmer-Lemeshow test. Two prediction models (pre-/intraoperative and early postoperative augmented) were evaluated, and sensitivity analyses excluding minor events were conducted.

## Results

### Distribution of pulmonary complications

**Figure 1:**
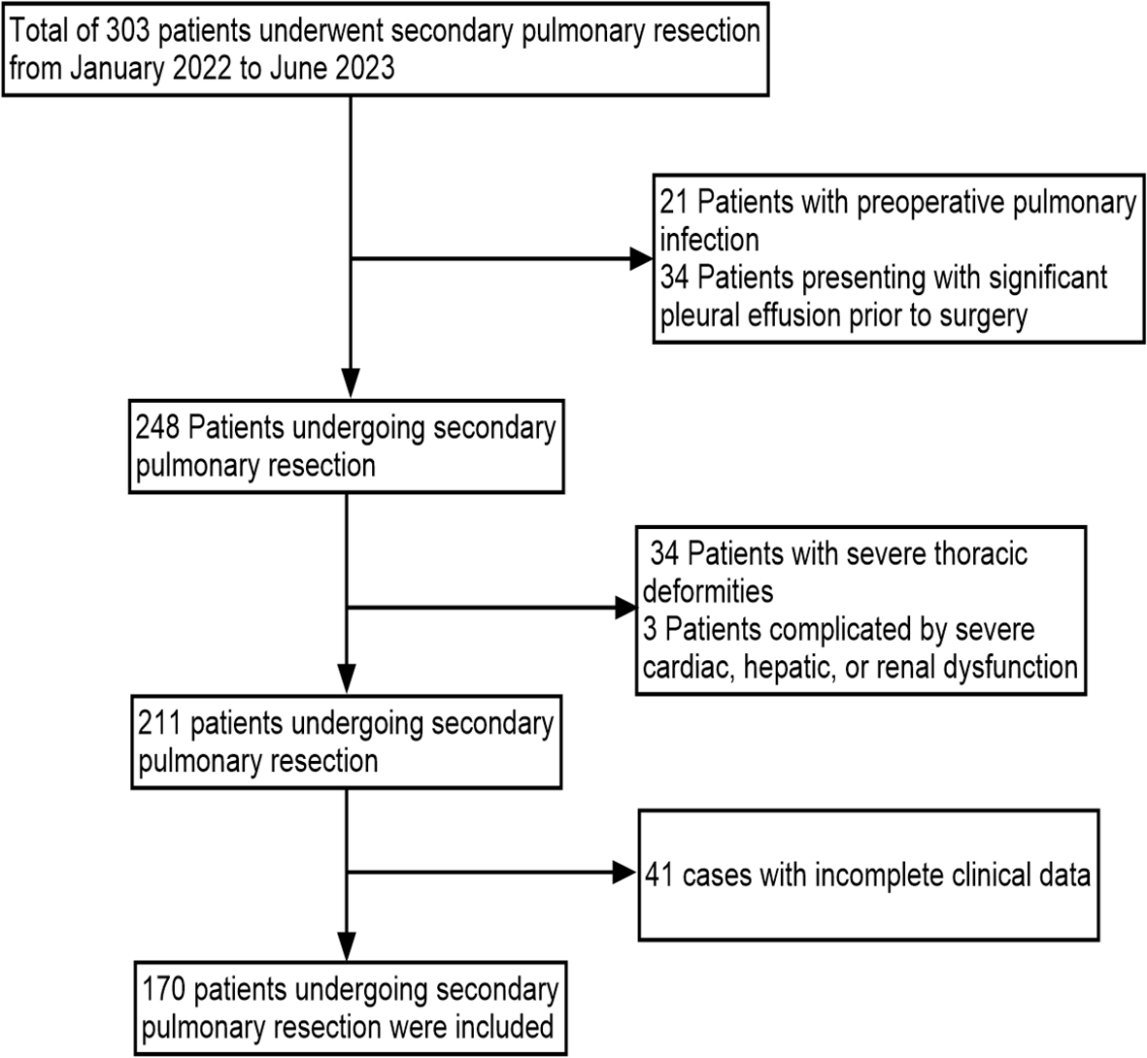
Patient screening flowchart.

A total of 170 patients scheduled for secondary pulmonary resection were included, comprising 65 males (38.2%) and 105 females (61.8%) (**Figure 1**). PPCs following secondary surgery included pulmonary infection, pneumothorax, pleural effusion, and pulmonary embolism, among others. Fifty-two patients (30.6%) experienced pulmonary complications (**Table 2**).

**Table 2:** Distribution of postoperative complications in patients (n=52).

PPCs	N(%)
pulmonary infection	32(18.8%)
pulmonary atelectasis	10(5.9%)
Prolonged air leak	9(5.3%)
Pulmonary embolism	2(1.2%)
Pneumothorax	4(2.4%)
Bronchospasms	2(1.2%)
Pleural effusion	5(2.9%)
Thoracic hemorrhage	2(1.2%)

### Comparison of baseline clinical characteristics and perioperative variables

Univariate analysis demonstrated significant differences between the PPC and non-PPC groups in gender, smoking index, preoperative PO2, duration of surgery, one-lung ventilation time, occurrence of moderate pain at 48 h postoperatively, closed drainage duration, FEV1%pred, LAV%, RFLV, PPO-FVC, PPO-FEV1, PPO-MVV, PPO-FEV1%pred, PPO-MVV%pred, and PPO-DLCO%pred (P < 0.05) (**Table 3**).

**Table 3:** Baseline characteristics and univariate comparisons between PPC and non-PPC groups.

Variables	PPCs(n=52)	Non-PPCs(n=118)	P
Genders, n (%)			0.005
males	28	37	
female	24	81	
Age (years)	57.75 ± 8.31	55.46 ± 9.43	0.132
BMI	23.50 ± 2.92	22.78 ± 2.48	0.099
smoking index	50 (0, 200)	0 (0,200)	0.040
cardiovascular disease, n (%)			0.783
yes	12(23.0%)	25(21.2%)	
no	40(77.0%)	93(78.8%)	
Preoperative PO2(mmHg)	69.61 ± 12.74	70.1 (66.4,74.0)	0.037
Preoperative PCO2(mmHg)	38.68 ± 4.12	38.92 ± 4.37	0.314
Use of plugging tubesn, n (%)	15(28.8%)	25(21.2%)	0.278
Use of Double-lumen bronchial tube, n (%)	37 (71.2%)	93 (78.8%)	0.278
Hemorrhage (ml)	50 (20,60)	50 (40,100)	0.555
Fluid administration (ml)	1000 (1000,1500)	1000 (1000,1500)	0.44
Duration of surgery (min)	150.4 ± 70.18	115.62 ± 53.20	0.001
one-lung ventilation time (min)	125.41 ± 70.18	90.58 ± 53.10	0.001
Moderate pain occurred 48H postoperatively (postoperative pain score ≥4), n (%)	34(65.4%)	25 (21.2%)	0.001
Closed Drainage Days	4 (3,6)	3 (2,4)	0.001
RV (L)	2.44 (2.03,3.36)	2.44(2.13,2.83)	0.07
TLC (L)	4.7 (4.15,5.89)	4.84(4.12,5.50)	0.101
RV/TLC (%)	52.92 (44.27,64.00)	51.34(45.7,59.06)	0.506
FEV1 (L)	2.17 ± 0.61	2.33 ± 0.61	0.222
FEV1%pred (%)	82.64 ± 16.27	96.90 ± 13.51	0.001
FVC (L)	2.74 ± 0.67	2.74 ± 0.70	0.776
FEV1/FVC (%)	80.59 ± 10.80	84.47 ± 10.66	0.388
MVV (L)	86.33 ± 26.03	91.75 ± 25.20	0.464
MVV%pred (%)	84.35 (66.08,101.13)	95.4(83.8,110.1)	0.137
DLCO(ml/min/mmHg)	8.47 ± 2.57	8.20 ± 2.03	0.485
DLCO%pred (%)	100.2 (88.83,114.73)	105.3(91.2,116.3)	0.942
FLV(ml)	4182.31 ± 1014.72	3961.25 ± 990.97	0.185
MLD(HU)	-820.87 ± 110.43	-830.08 ± 76.46	0.531
LAV(%)	34.39 ± 11.18	27.92 ± 12.46	0.002
RFLV(ml)	687.00 (433.09, 991.44)	318.80(206.90,482.35)	0.001
PPO-RV	2.33 ± 1.10	2.28 ± 0.70	0.708
PPO-TLC	4.026(3.47,4.54)	4.25(3.79,4.96)	0.1
PPO-FVC	2.21 ± 0.57	2.48 ± 0.65	0.010
PPO-FEV1	1.69(1.37.2.23)	1.99(1.68,2.43)	0.002
PPO-MVV	64.87(59.58, 86.67)	77.80(65.11,98.18)	0.005
PPO-DLCO	6.87 ± 2.12	7.41 ± 1.95	0.108
PPO-FEV1%pred	68.05 ± 17.55	85.85 ± 14.26	0.001
PPO-MVV%pred	70.36(56.91, 84.11)	83.97(72.17,95.90)	0.001
PPO-DLCO%pred	89.47 ± 25.70	93.69 ± 20.40	0.012

### Binary logistic regression analysis results

Binary logistic regression analysis indicated that FEV1%pred (OR = 0.86, 95% CI = 0.789–0.938), LAV% (OR = 1.057, 95% CI = 1.011–1.106), PPO-FEV1%pred (OR = 0.86, 95% CI = 0.789–0.938), and one-lung ventilation time (OR = 1.009, 95% CI = 1.000–1.019) were pre-/intraoperative independent predictors of overall PPC occurrence (**Table 4**). In addition, moderate pain at 48 h postoperatively was an early postoperative risk marker that remained independently associated with PPCs (OR = 12.32, 95% CI = 3.903–38.898).

**Table 4:** Binary logistic analysis of the predictors of PPC in patients undergoing surgery for secondary lung cancer.

Variables	Univariate				Multivariate	
	OR	95%CI	P	OR	95%CI	P
Genders	2.554	1.307∼4.990	0.006	0.721	0.153∼3.409	0.68
Smoking situation	1.002	1∼1.0003	0.063	0.708	0.237∼2.115	0.537
Preoperative PO2(mmHg)	0.985	0.953∼1.019	0.384	0.967	0.914∼1.023	0.242
one-lung ventilation time(min)	1.009	1.004∼1.015	<0.001	1.009	1.000∼1.019	0.046
Moderate pain occurred 48H postoperatively (postoperative pain score ≥4)	0.142	0.069∼0.293	<0.001	12.32	3.903∼38.898	0.001
Closed Drainage Days	1.194	1.031∼1.381	0.018	1.246	0.973∼1.597	0.081
FEV1%pred	0.953	0.930∼0.977	<0.001	0.86	0.789∼0.938	0.004
LAV%	1.047	1.016∼1.078	0.02	1.057	1.011∼1.106	0.015
PPO-FEV1	0.782	0.464∼1.318	0.356	0.889	0.116∼6.836	0.91
PPO-MVV	0.997	0.985∼1.009	0.639	0.972	0.929∼1.017	0.22
PPO-FEV1%pred	0.924	0.898∼0.951	<0.001	0.86	0.789∼0.938	0.001
PPO-MVV%pred	0.961	0.942∼0.980	<0.001	1.003	0.986∼1.020	0.77
PPO-DLCO%pred	0.980	0.962∼0.996	0.014	1	0.977∼1.023	0.97

### ROC curve analysis

**Figure 2:**
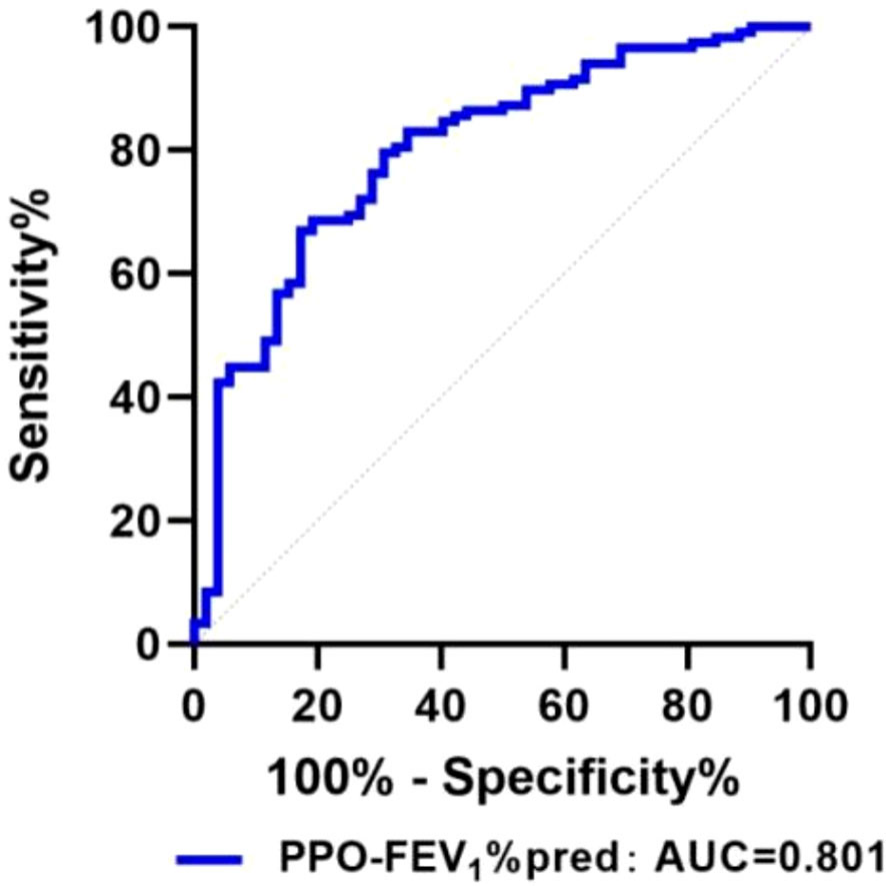
The ROC curve of PPO-FEV1%pred for predicting PPCs.

ROC curve results demonstrated that an FEV1%pred threshold of less than 83.95% optimally predicted PPCs, as identified by maximal Youden’s index. For FEV1%pred, the AUC was 0.683 (95% CI = 0.594∼0.771), accompanied by a sensitivity of 84.70% and specificity of 48.10% (**Table 5**). Likewise, an LAV% threshold exceeding 32.65% yielded an AUC of 0.653 (95% CI = 0.566–0.740), sensitivity of 59.6%, and specificity of 68.6% (**Table 5**). Furthermore, PPO-FEV1%pred below 79.45% predicted PPC occurrence with an AUC value of 0.801 (95% CI = 0.728–0.873), sensitivity of 82.7%, and specificity of 49.6% (**Table 5**; **Figure 2**). Comparisons of AUCs confirmed that PPO-FEV1%pred exhibited the most robust diagnostic accuracy, reaching statistical significance (P < 0.05) (**Table 5**).

**Table 5:** Comparison of ROC curves by the indicators.

Variables	AUC	P	95% CI	Cut off	Sensitivity	Specificity
FEV1%pred	0.683	0.001	0.594∼0.771	83.95	84.70	48.10
PPO-FEV1%pred	0.801	0.001	0.728∼0.873	79.45	82.70	49.60
LAV%	0.653	0.001	0.566∼0.740	32.65	59.60	68.60
one-lung ventilation time	0.646	0.002	0.556∼0.736	140	38.50	82.20

### Model building and internal validation and calibration

#### Model 1: pre-/intraoperative model

**Figure 3:**
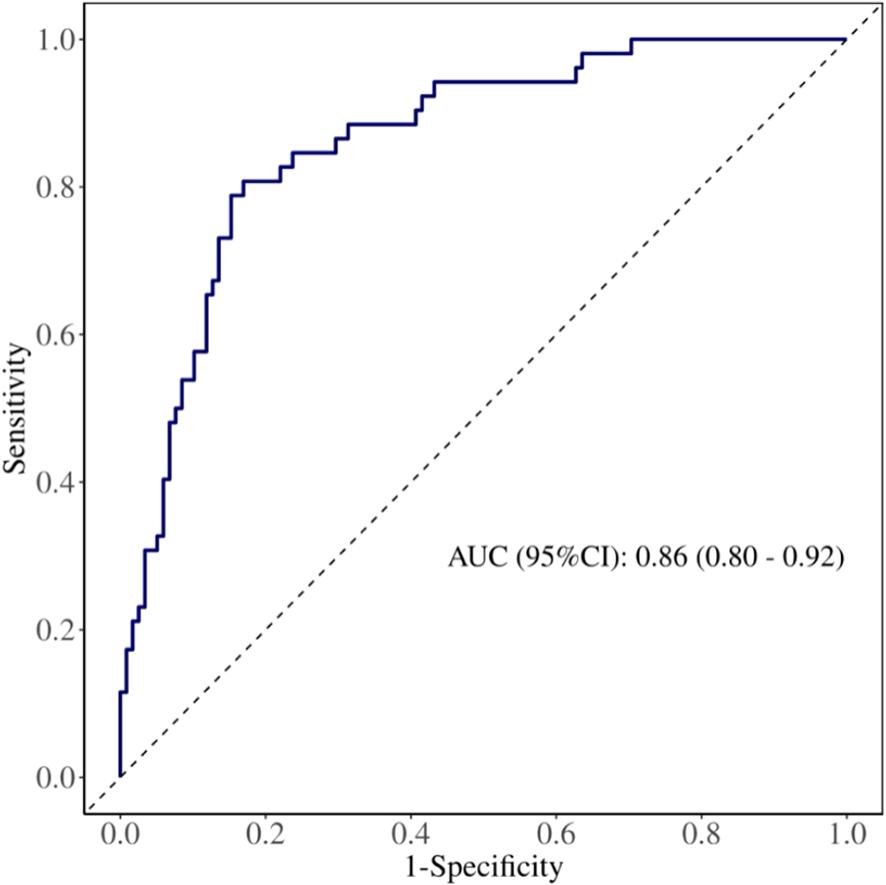
ROC of pre-/intraoperative model for predicting PPCs.

**Figure 4:**
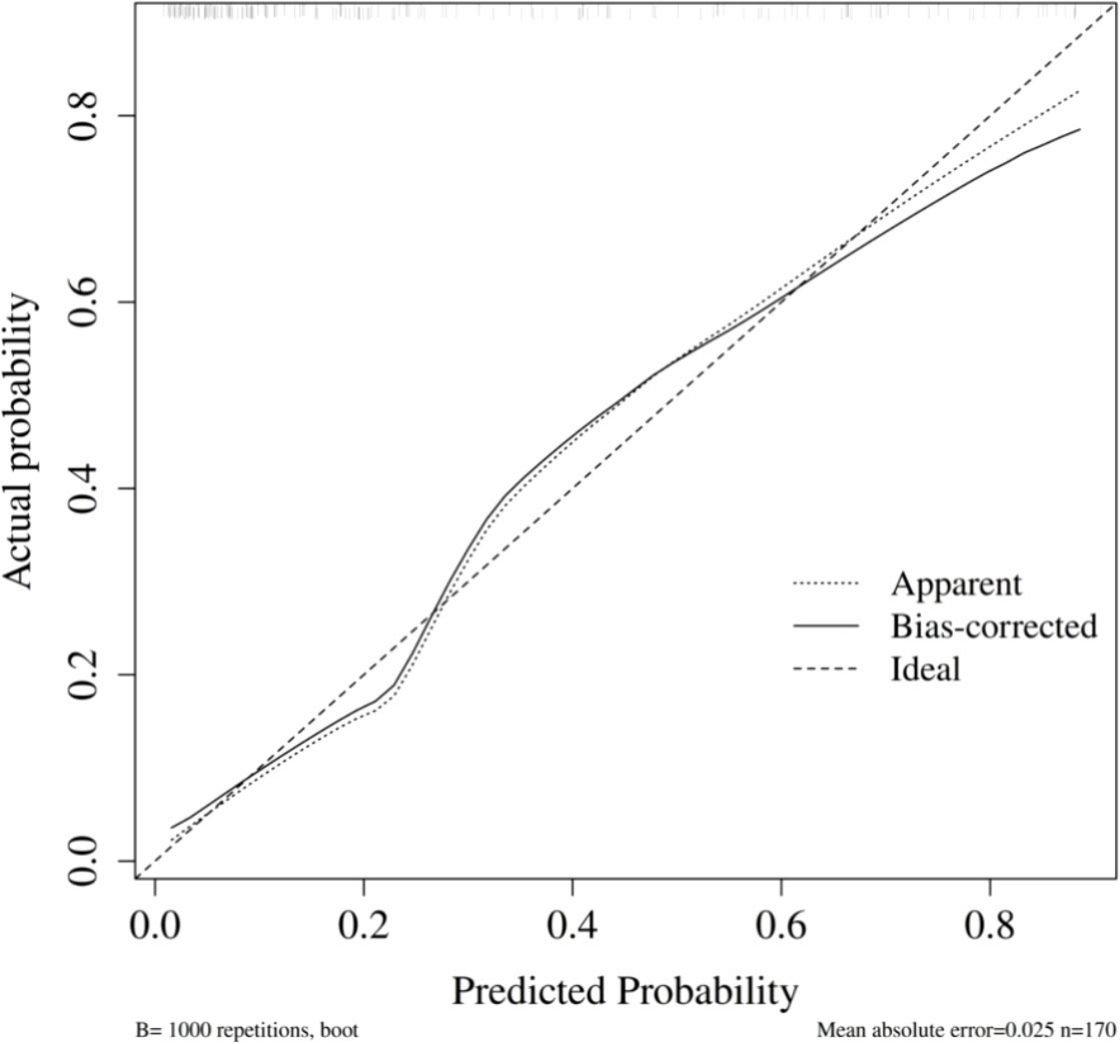
Calibration curve of pre-/intraoperative model for predicting PPCs.

Internal validation was conducted using the bootstrap method (repeated sampling 1,000 times). The model’s area under the ROC curve (AUC) was 0.86 (95% CI: 0.80–0.92) (**Figure 3**). Bootstrap internal validation results indicated an optimistically calibrated AUC of 0.86, suggesting the model possesses good discriminatory capability. The optimally adjusted calibration slope was 0.93, approaching unity. Both the apparent curve and the bias-corrected curve generally approximated the ideal line, indicating overall consistency between predicted probabilities and actual risk occurrence (**Figure 4**). The Hosmer-Lemeshow test suggested acceptable model fit (χ²=7.670, P = 0.466).

#### Model 2: early postoperative augmented model

**Figure 5:**
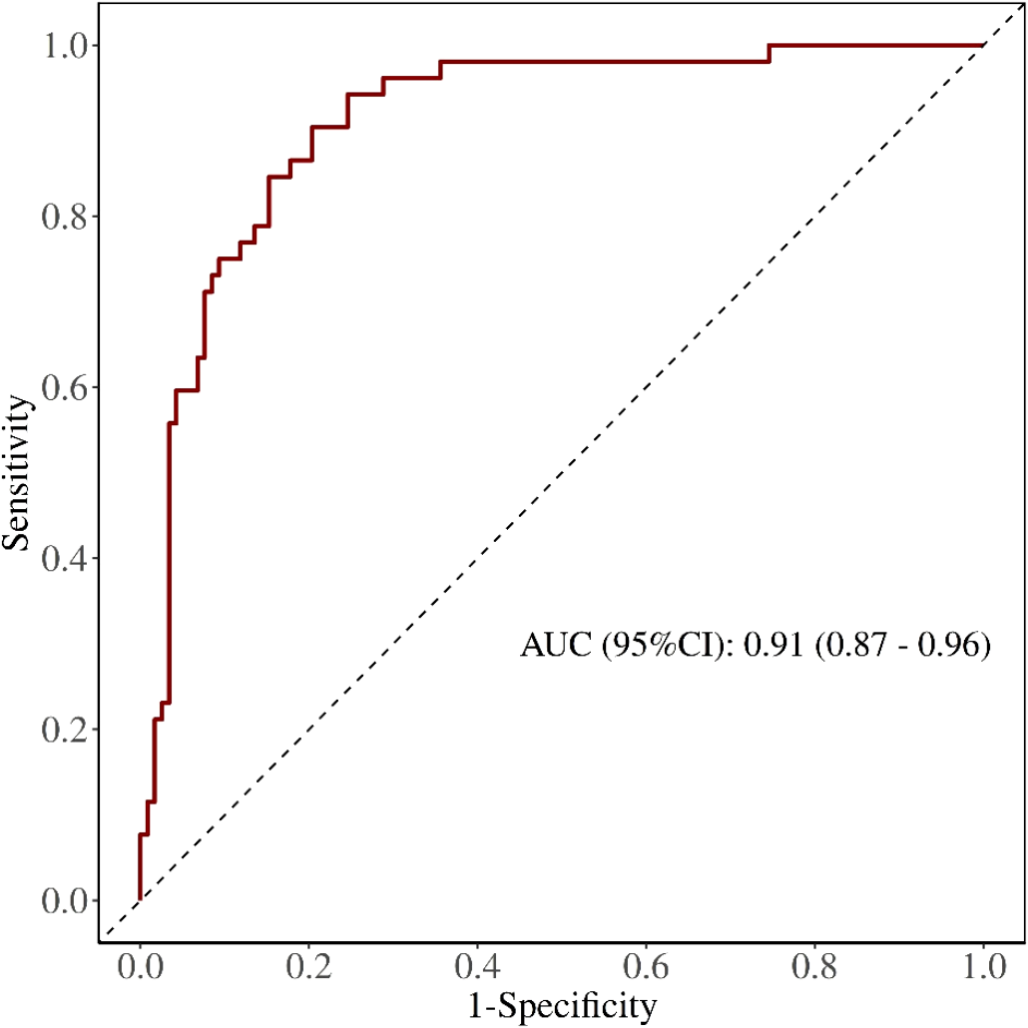
ROC of early postoperative augmented model for predicting PPCs.

**Figure 6:**
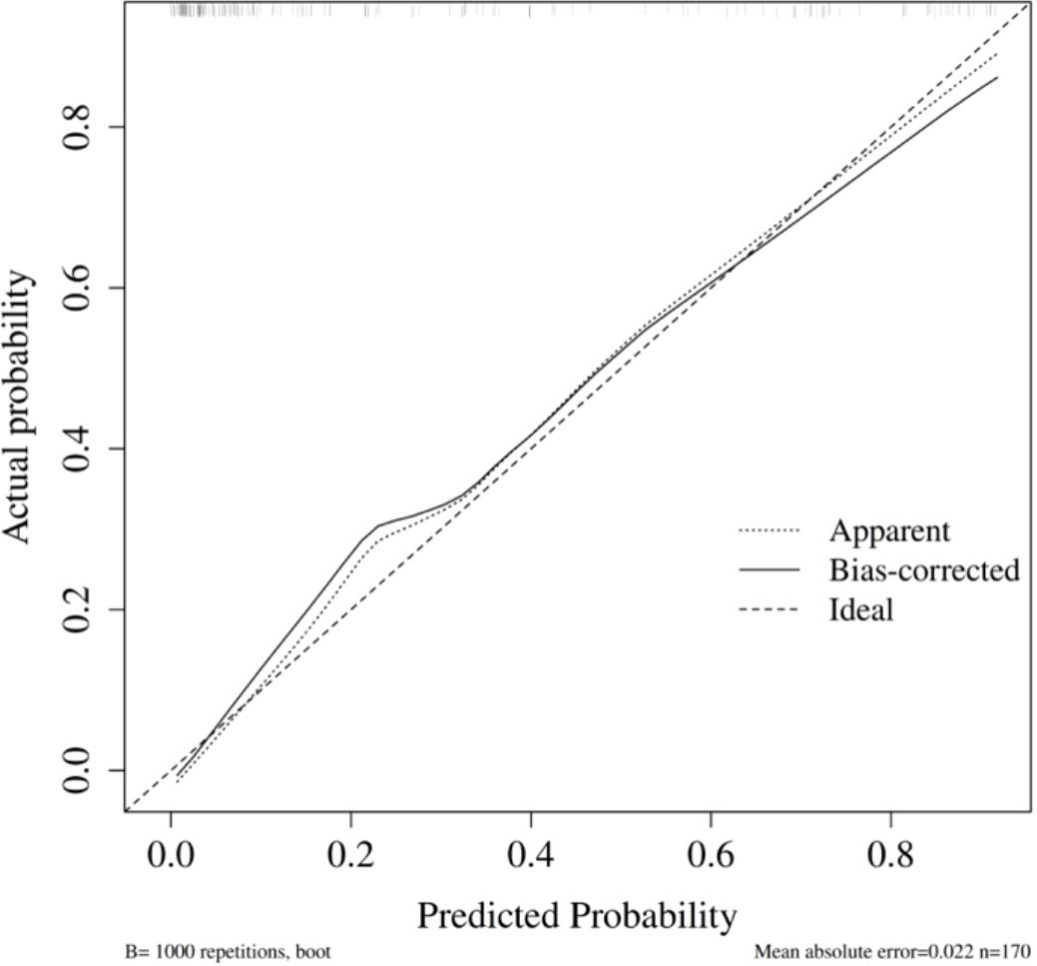
Calibration curve of early postoperative augmented model for predicting PPCs.

Internal validation was conducted using the bootstrap method (1,000 repeated samples). The model’s area under the ROC curve (AUC) was 0.91 (95% CI: 0.87–0.96) (**Figure 5**). Bootstrap internal validation results indicated an optimistically calibrated AUC of 0.91, suggesting the model possesses good discriminatory capability. The optimally adjusted calibration slope was 0.90, approaching unity. Both the apparent curve and the bias-corrected curve generally approximated the ideal line, indicating overall consistency between predicted probabilities and actual risk occurrence (**Figure 6**). The Hosmer-Lemeshow test suggested acceptable model fit (χ²=10.984, P = 0.203).

### Sensitivity analysis excluding minor events

Sensitivity analysis (using a subset of cases after excluding milder events) revealed that the ROC curve results indicated an optimal discriminatory threshold for PPCs at an FEV1%pred below 83.95%, where the Youden index was maximized. The AUC for FEV1%pred was 0.661 (95% CI = 0.563–0.759), corresponding to a sensitivity of 42.86% and specificity of 84.82% (**Table 6**). Similarly, for LAV% > 34.95%, the AUC was 0.670 (95% CI = 0.574–0.766), with sensitivity 59.52% and specificity 72.32% (**Table 6**). Furthermore, PPO-FEV1%pred below 78.94% predicted PPC occurrence, yielding an AUC of 0.780 (95% CI = 0.696–0.863), sensitivity 80.95%, and specificity 67.86% (**Table 6**). When one-lung ventilation time exceeded 140 minutes, the AUC was 0.676 (95% CI = 0.578–0.774), with a sensitivity of 45.24% and specificity of 82.14% (**Table 6**). Overall comparisons demonstrated that PPO-FEV1%pred retained the strongest discriminatory capability in sensitivity analyses (P<0.001) (**Table 6**).

**Table 6:** Comparison of ROC curves by the indicators (minor PPCs excluded).

Variable	AUC	P	95%CI	cutoff	Sensitivity	Specificity
FEV1%pred	0.661	0.001	0.563∼0.759	83.95	42.86	84.82
PPO-FEV1%pred	0.780	<0.001	0.696∼0.863	78.94	80.95	67.86
LAV%	0.670	<0.001	0.574∼0.766	34.95	59.52	72.32
One-lung ventilation time	0.676	<0.001	0.578∼0.774	140	45.24	82.14

#### Model 1: pre-/intraoperative model

**Figure 7:**
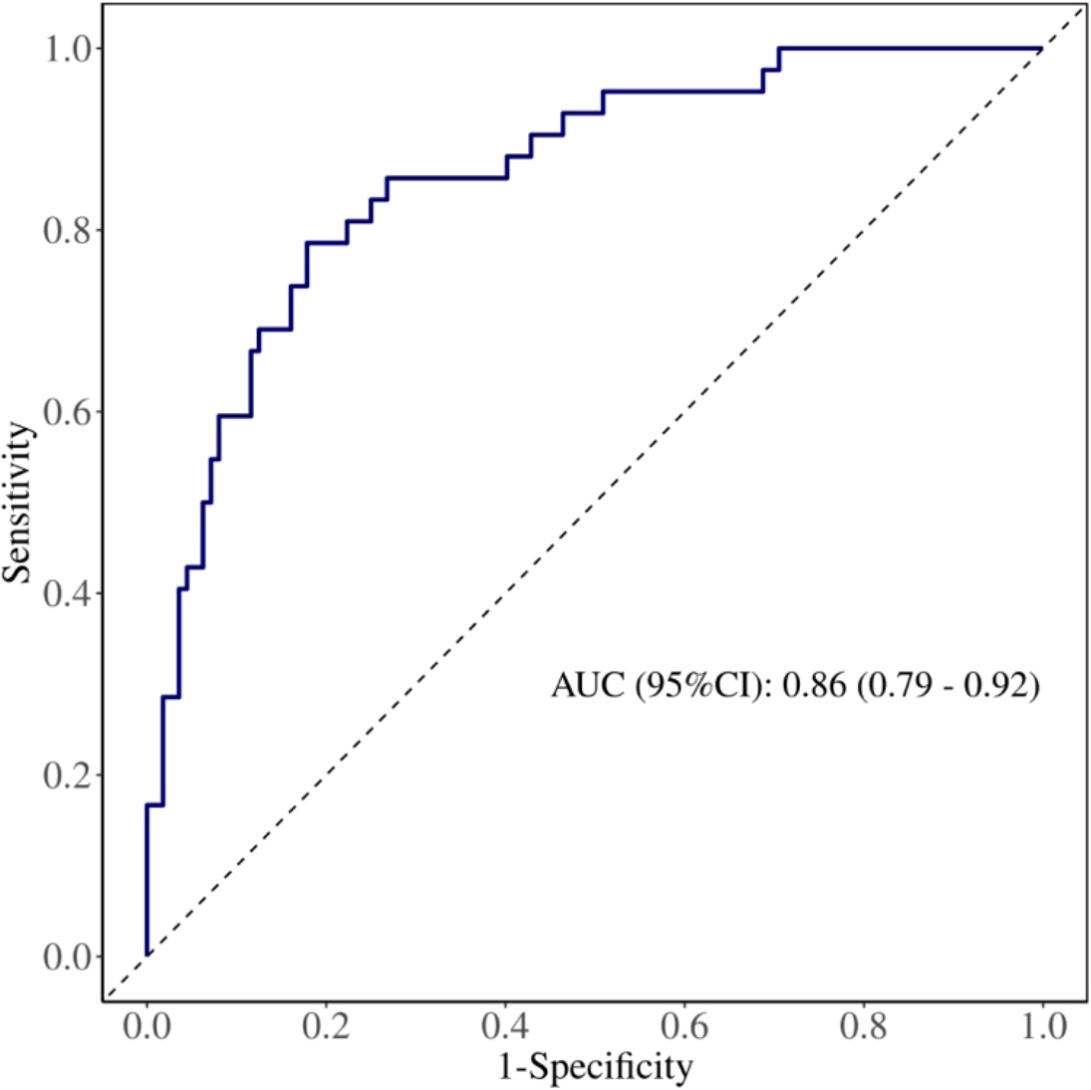
ROC of the Pre-/intraoperative model in sensitivity analysis (minor PPCs excluded).

**Figure 8:**
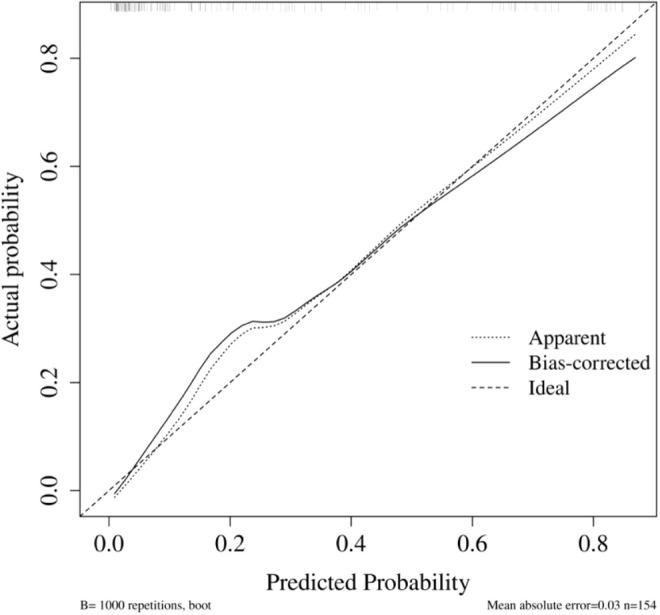
Calibration of the pre-/intraoperative model sensitivity analysis (minor PPCs excluded).

The model’s area under the ROC curve (AUC) was 0.86 (95% CI: 0.79–0.92) (**Figure 7**). Bootstrap internal validation results indicated an optimistically calibrated AUC of 0.86, The optimally adjusted calibration slope was 0.91, approaching unity. Both the apparent curve and the bias-corrected curve generally approximated the ideal line, indicating overall consistency between predicted probabilities and actual risk occurrence (**Figure 8**). The Hosmer-Lemeshow test suggested acceptable model fit (χ²=5.187, P = 0.738).

#### Model 2: early postoperative augmented model

**Figure 9:**
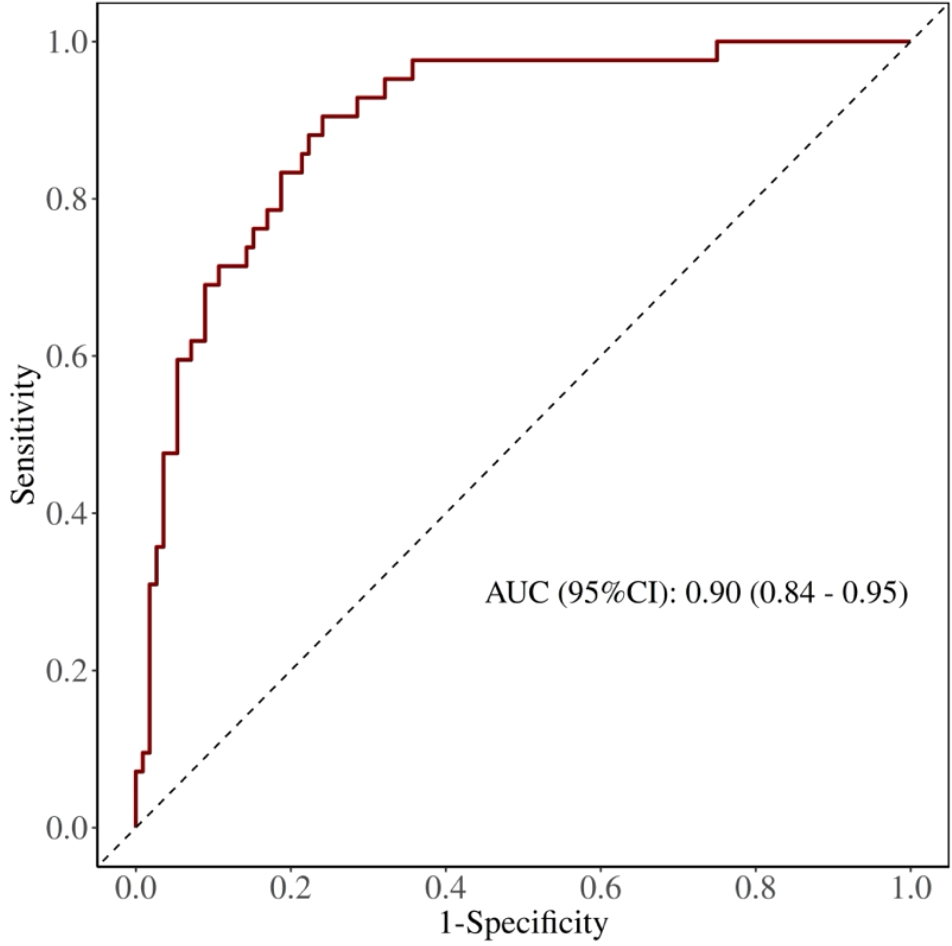
ROC of the early postoperative augmented model in sensitivity analysis (minor PPCs excluded).

**Figure 10:**
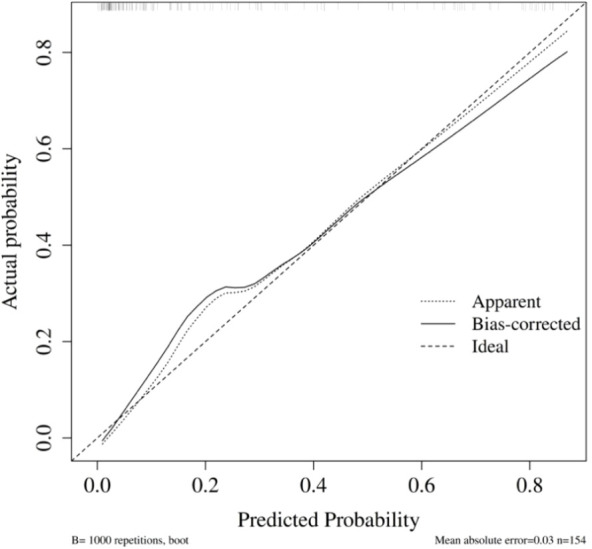
Calibration of the early postoperative augmented model in sensivity analysis (minor PPCs excluded).

The model’s area under the ROC curve (AUC) was 0.90 (95% CI: 0.84–0.95) (**Figure 9**). Bootstrap internal validation results indicated an optimistically calibrated AUC of 0.90, The optimally adjusted calibration slope was 0.88, approaching unity. Both the apparent curve and the bias-corrected curve generally approximated the ideal line, indicating overall consistency between predicted probabilities and actual risk occurrence (**Figure 10**). The Hosmer-Lemeshow test suggested acceptable model fit (χ²=7.533, P = 0.480).

## Discussion

With the widespread adoption of early lung cancer screening, an increasing number of MPLC cases are being identified (3). Surgical resection remains the mainstay of treatment for patients with MPLC (4). However, insufficient respiratory reserve in patients undergoing secondary surgery with already impaired lung function may result in severe PPCs and mortality. Currently, effective methods for predicting PPCs are lacking. Identifying relevant clinical and imaging features to predict PPCs is therefore crucial. In this study, FEV1%pred, one-lung ventilation time, LAV%, and PPO-FEV1%pred were identified as pre-/intraoperative independent predictors of overall PPC occurrence. Moderate pain at 48 h postoperatively was independently associated with PPCs and should be interpreted as an early postoperative risk marker. These variables demonstrated good predictive value. PPO-FEV1%pred showed the highest predictive efficacy. These findings provide a reference for the clinical management of patients with MPLC.

Linden et al. (18) conducted a retrospective study of 186 MPLC patients and reported a 30-day postoperative mortality rate of 11.0% and a complication rate of 19.0%. Hamaji et al. (19) reported a complication rate of 29% among 161 patients after secondary surgery. The PPC rate in this study (30.6% among 170 patients) is slightly higher than in previous reports, potentially due to the majority of patients residing at high altitudes for extended periods. High altitude is defined as a geographic elevation >1500 m above sea level. Although no universally accepted definition exists, the literature widely recognizes this elevation as a physiological demarcation (20–23). High altitude significantly affects the respiratory system, as decreased atmospheric pressure reduces oxygen partial pressure, leading to compensatory cardiopulmonary adaptations to tolerate hypoxia and maintain tissue oxygenation (24). Thus, patients living at high altitudes have diminished pulmonary reserves preoperatively, rendering them more susceptible to PPCs (25).

Alessandro Brunelli et al. reported that DLCO and its predicted postoperative value are critical predictors of adverse perioperative outcomes (26). Other studies have similarly shown that CPET-derived variables correlate with postoperative outcomes after lung resection (27). In recent years, the focus on PPC assessment has shifted towards predicting postoperative pulmonary function after the first lung resection. Accurate assessment of postoperative pulmonary function after the first lung resection is critical for reducing PPCs after secondary surgery and improving postoperative quality of life. The most frequently utilized clinical method to predict postoperative pulmonary function after the first lung resection currently is anatomical segmentation, computed as follows: postoperative pulmonary function index = preoperative pulmonary function index × [1 - (resected lung segments/total lung segments)] (28). However, functional lung tissue changes occur across all segments after first surgery. Richard et al. (29) conducted a pooled analysis of thoracic surgery data from 27 hospitals across 14 countries, concluding that preoperative PFTs second surgery did not accurately predict postoperative complication risks after secondary surgery. Therefore, this formula is unsuitable for such cases.

Recent studies have used three-dimensional CT (3D-CT) volumetry to estimate postoperative lung function, demonstrating strong correlations with measured postoperative spirometric parameters (30–32). Building on these contemporary CT-based methods, our study focuses on predicting PPC risk following secondary surgery in MPLC. The QCT-derived indices in our model represent lung parenchymal characteristics, potentially complementing volumetric prediction methods. QCT identifies functional lung tissue based on CT attenuation values, accurately assessing lung function regionally. Its value in predicting postoperative lung function has increasingly been recognized. The density range for functional lung tissue assessed by QCT is between -910 and -600 HU (33). QCT calculates the volume of functional lung tissue. Lung tissue with density values greater than -600 HU indicates reduced ventilation, representing restrictive dysfunction, while tissue with density values below -910 HU indicates excessive ventilation, representing obstructive dysfunction. The lung volume within this density range is termed FLV. The volume of resected lobes within this density interval is termed RFLV. Thus, the predicted postoperative lung function (PPO-value) is calculated as preoperative lung function index × [1 - (RFLV/FLV)].

In the current study, PPO-values derived using QCT demonstrated good performance in predicting PPCs among patients undergoing secondary surgery. Specifically, QCT-derived PPO-FEV1%pred showed strong predictive performance for PPCs when values were <79.45% in our high-altitude cohort. Clinically, this cutoff may inform perioperative risk stratification. Patients below this threshold may be flagged as higher risk, warranting intensified perioperative optimization and monitoring. Such measures include individualized anesthetic and ventilatory strategies intraoperatively, early postoperative respiratory physiotherapy, enhanced PPC surveillance, and a lower threshold for ICU-level observation when clinically indicated. However, because these ROC-derived cutoff values were determined using the Youden index in a single-center cohort, they should be interpreted as exploratory thresholds requiring external validation before routine clinical implementation, rather than serving as rigid criteria for surgical candidacy.

LAV% is a parameter used to assess lung density via QCT analysis. It represents the percentage of total lung volume with low-density attenuation areas below -950 HU. A higher LAV% indicates a greater area of emphysema and worse lung function. Consistent with our findings, patients with higher LAV% were more prone to PPCs. Therefore, preoperative LAV% and emphysema status should receive careful attention. Moderate pain at 48 h postoperatively exhibited a high odds ratio in our multivariable model. VAS scores indicate postoperative pain severity, with higher scores reflecting more intense pain. Acute and chronic postoperative pain are well-recognized complications after thoracic surgery and can substantially affect quality of life and healthcare utilization (34). Niraj et al. (35) reported that moderate-to-severe acute postoperative pain may impair residual lung expansion, potentially contributing to pulmonary morbidity by limiting deep breathing and effective coughing. In our cohort, moderate pain at 48 h was independently associated with PPCs within 30 days, suggesting postoperative pain may serve as an early risk marker for pulmonary complications. Clinically, this finding supports close postoperative respiratory monitoring and optimized analgesia in patients with higher pain levels. However, this association should be interpreted as predictive rather than definitively causal.

This single-center study has limitations, including potential selection bias due to limited patient diversity. The accuracy and comprehensiveness of the data depended on medical record completeness and detail. In addition, perioperative and anesthetic management protocols are institution-specific. Thus, unmeasured differences in ventilation strategies, analgesia, fluid management, and postoperative respiratory care may confound associations and limit generalizability. Although we applied a parsimonious modeling strategy, overfitting or overly optimistic model performance cannot be excluded, particularly given the use of backward elimination for variable selection and the lack of external validation. Therefore, validation in independent cohorts is warranted. The cutoff values identified in this study were derived from our cohort using the Youden index and should be considered exploratory. Given the single-center retrospective design and potential variability in case mix and quantitative CT workflows, these thresholds may lack generalizability and require external validation and recalibration before routine clinical adoption. Since many patients in our cohort resided at high altitude, the ROC-derived cutoff values might be altitude- and center-specific, further emphasizing the need for validation in low-altitude cohorts. Moreover, PPCs represent a heterogeneous composite endpoint comprising complications of varying mechanisms and severity; thus, predictors such as PPO-FEV1%pred and LAV% may not equally predict all PPC subtypes. In addition, we did not apply a standardized severity grading system for postoperative complications. Future prospective studies with larger sample sizes and standardized severity grading systems should validate predictive performance across different PPC subtypes. Finally, we assessed model discrimination and calibration in the current dataset, including bootstrap internal validation and calibration analysis with goodness-of-fit testing; nevertheless, external validation and potential recalibration in independent cohorts remain necessary.

## Conclusion

This study identified FEV1%pred, one-lung ventilation time, LAV%, and PPO-FEV1%pred as pre-/intraoperative independent predictors of overall PPC occurrence. Moderate pain at 48 h postoperatively was an early postoperative risk marker that remained independently associated with PPCs. PPO-FEV1%pred demonstrated the highest diagnostic accuracy for predicting PPCs after secondary pulmonary resection, facilitating personalized clinical decision-making and patient management.

## Data Availability

The raw data supporting the conclusions of this article will be made available by the authors, without undue reservation.
